# A Phthalimide Derivative That Inhibits Centrosomal Clustering Is Effective on Multiple Myeloma

**DOI:** 10.1371/journal.pone.0038878

**Published:** 2012-06-25

**Authors:** Hirokazu Shiheido, Fukiko Terada, Noriko Tabata, Ichigo Hayakawa, Nobutaka Matsumura, Hideaki Takashima, Yoko Ogawa, Wenlin Du, Taketo Yamada, Mitsuru Shoji, Takeshi Sugai, Nobuhide Doi, Shiro Iijima, Yutaka Hattori, Hiroshi Yanagawa

**Affiliations:** 1 Department of Biosciences and Informatics, Keio University, Hiyoshi, Kohoku-ku, Yokohama, Japan; 2 Clinical Physiology and Therapeutics, Faculty of Pharmacy, Keio University, Minato-ku, Tokyo, Japan; 3 Department of Pathology, School of Medicine, Keio University, Shinjuku-ku, Tokyo, Japan; 4 Organic and Biocatalytic Chemistry, Faculty of Pharmacy, Keio University, Minato-ku, Tokyo, Japan; University of Illinois at Chicago, United States of America

## Abstract

Despite the introduction of newly developed drugs such as lenalidomide and bortezomib, patients with multiple myeloma are still difficult to treat and have a poor prognosis. In order to find novel drugs that are effective for multiple myeloma, we tested the antitumor activity of 29 phthalimide derivatives against several multiple myeloma cell lines. Among these derivatives, 2-(2,6-diisopropylphenyl)-5-amino-1*H*-isoindole-1,3- dione (TC11) was found to be a potent inhibitor of tumor cell proliferation and an inducer of apoptosis *via* activation of caspase-3, 8 and 9. This compound also showed *in vivo* activity against multiple myeloma cell line KMS34 tumor xenografts in ICR/SCID mice. By means of mRNA display selection on a microfluidic chip, the target protein of TC11 was identified as nucleophosmin 1 (NPM). Binding of TC11 and NPM monomer was confirmed by surface plasmon resonance. Immunofluorescence and NPM knockdown studies in HeLa cells suggested that TC11 inhibits centrosomal clustering by inhibiting the centrosomal-regulatory function of NPM, thereby inducing multipolar mitotic cells, which undergo apoptosis. NPM may become a novel target for development of antitumor drugs active against multiple myeloma.

## Introduction

Multiple myeloma is one of the hematopoietic organ tumors which is characterized by the monoclonal proliferation of malignant plasma cells, resulting in appearance of serum or urinary monoclonal protein [1]–[3]. Although treatments include chemotherapy with melphalan, prednisolone or cyclophosphamide, as well as hematopoietic stem cell transplantation [4]–[8], most patients become refractory to the therapy and this leads to a fatal outcome. A group of high-risk patients is exclusively poorly responsive, with short survival. Tumor cells derived from high-risk patients have deletion of chromosome 17 (del 17), on which the p53 tumor suppressor gene is located, deletion of chromosome 13 or chromosomal translocation t(4;14) accompanied with constitutive activation of FGFR-3 on chromosome 4 [9]–[11]. Therefore, development of novel drugs which are active against multiple myeloma cells with these high-risk chromosomal or genetic alterations is necessary to improve the prognosis. Moreover, elucidation of the mechanisms of growth suppression of multiple myeloma cells is expected to improve our understanding of the molecular pathogenesis of multiple myeloma. Hence, our aim in this study was to find a novel anti-tumor drug for multiple myeloma and to elucidate its molecular mechanism of action.

Drugs such as thalidomide, lenalidomide, and bortezomib show anti-tumor effect on multiple myeloma and have received much attention in recent years [12]–[14]; however, even these potent drugs are of limited value in high-risk cases [15]. Here, we tested the growth-inhibitory effect of 29 phthalimide derivatives, which are similar in structure to thalidomide, against several multiple myeloma cell lines including those with del 17 or t(4;14). We found that one of these derivatives, 2-(2,6-diisopropylphenyl)-5-amino-1*H*-isoindole-1,3-dione (TC11), showed potent inhibition of tumor cell proliferation both *in vitro* and *in vivo*, and induced apoptosis. Furthermore, we utilized mRNA display [16] to identify nucleophosmin 1 (NPM/B23), a nucleolar phosphoprotein [17], [18], as a target of TC11 for inducing apoptosis of tumor cells. Inhibiting the function of NPM led to induction of multipolar mitoses by blocking centrosomal clustering, and this leads to cell death. Our results thus indicate that NPM could be a novel target for development of anticancer drugs to overcome the poor prognosis of high-risk multiple myeloma patients.

## Results

### Screening of Synthetic Phthalimide Derivatives to Identify Compounds Inhibiting Multiple Myeloma Cell Proliferation

Initially, we tested 29 phthalimide derivatives, TC1-29, each of which has various functional groups (Figure S1), for activity to inhibit proliferation of multiple myeloma KMS34 cells with t(4;14) and del 17. In the first screening, we performed cell proliferation assay to examine inhibitory activity. KMS34 cells were incubated with 50 µM of each compound for 0, 24, 48 or 72 h and the cell viability was determined with WST-1 assay. Nine compounds, TC8, TC9, TC10, TC11, TC12, TC13, TC14, TC15, and TC16, showed growth-inhibitory activity, while the others were inactive (Figure S2A). Therefore, we further examined the active compounds.

To identify the most potent compound, we investigated the ability of the hit compounds from the first screening to inhibit proliferation or to induce apoptosis of several multiple myeloma cell lines. In the second screening, KMM1, KMS11, KMS27, KMS34 and RPMI8226 cells were incubated with 0–50 µM TC8, TC9, TC10, TC11, TC12, TC13, TC14, TC15, and TC16. One arm of chromosome 17 is deleted in KMM1, KMS11 and KMS34 cells, and KMS11 and KMS34 also show t(4;14) (our unpublished data). In this screening, TC11 and TC13 showed potent activity against all cell lines tested, with IC_50_ values of 4–8 µM and 4–11 µM, respectively (Table 1). Furthermore, we tested the apoptosis-inducing activity of TC11 and TC13. The results indicated that TC11 required a lower concentration or a shorter treatment time to induce apoptosis of several multiple myeloma cell lines (Figure S2B). Thus, we identified TC11 as the most potent compound, capable of inhibiting multiple myeloma cell lines with high-risk chromosomal changes, t(4;14) and del 17.

**Table 1:** IC_50_ values (µM) of thalidomide derivatives for inhibiting proliferation of multiple myeloma cell lines.

Cell line	Compound									
	TC8	TC9	TC10	TC11	TC12	TC13	TC14	TC15	TC16	Thalidomide
KMM1	>50	>50	>50	7	18	5	32	>50	>50	>50
KMM11	>50	>50	>50	6	14	4	7	>50	>50	>50
KMS27	>50	>50	>50	8	25	8	34	>50	>50	>50
KMS34	>50	>50	25	4	16	5	14	>50	>50	>50
RPMI8226	>50	>50	>50	7	26	11	24	>50	>50	>50

**Figure 1:**
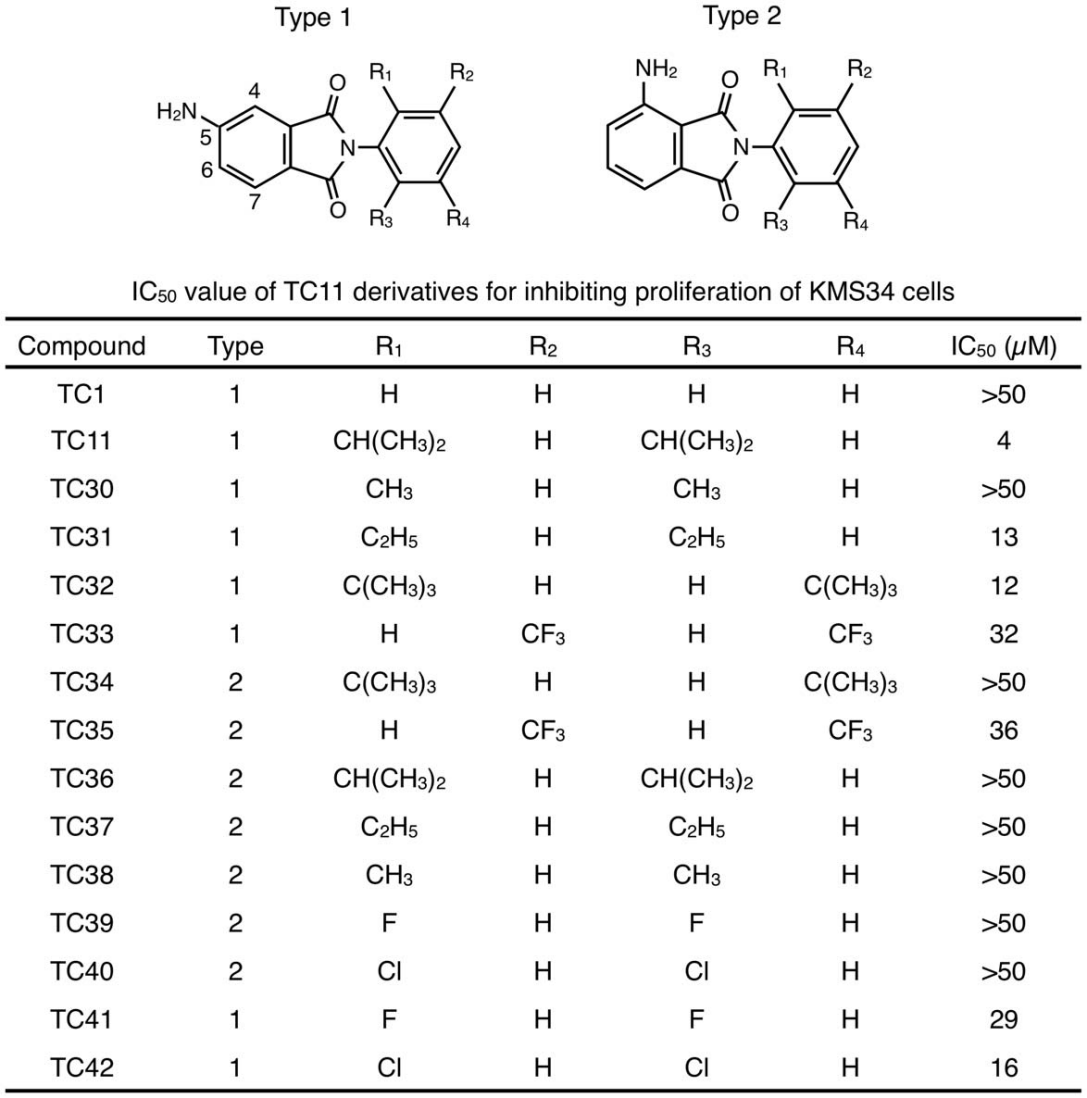
IC_50_ values of TC11 derivatives for inhibiting proliferation of KMS34 cells. KMS34 cells (1×10^4^ cells/well) in 96-well plate were incubated with 0–50 µM of indicated compound for 72 h. Then cell viability was determined with WST-1 assay.

To optimize the potency of TC11, we further synthesized TC11 derivatives TC30-42 (Figure 1) and tested them. KMS34 cells were incubated with 0–50 µM TC11 derivatives for 72 h and the cell viability was assayed. First of all, to examine the importance of the position of the amino group in the phthalimide moiety, we synthesized 7 derivatives with a 4-amino group (Type 2 in Figure 1) instead of a 5-amino group (Type 1 in Figure 1). Almost all derivatives with a 4-amino group lacked anti-tumor activity, suggesting that the 5-amino group of TC11 is important for anti-tumor activity. We next replaced functional groups of the phenyl ring of TC11 [R1–R4] with others [H, CH_3_, C_2_H_5_, CH(CH_3_)_2_, C(CH_3_)_3_, CF_3_, F or Cl]. Substitution of R1 and R4 with a hydrogen atom (TC1) dramatically decreased the anti-tumor activity. Furthermore, 5-amino phthalimide derivatives with methyl groups (TC30), ethyl groups (TC31), fluorine atoms (TC41) or chlorine (TC42) atoms at the R1 and R4 positions showed decreased anti-tumor activity. A 5-amino phthalimide derivative (TC32) with sterically bulky tertiary butyl groups at the R1 and R3 positions of the phenyl ring showed much the same anti-tumor activity as TC31 with ethyl groups at the R1 and R4 positions of the phenyl ring. These results indicate that the phenyl ring requires relatively large functional groups, such as isopropyl groups, at both R1 and R4. These findings suggested that TC11 with an amino group at the 5-position of phthalimide moiety and isopropyl groups at both *ortho* positions of the phenyl ring is the most potent compound, in terms of growth inhibition of multiple myeloma cells.

### TC11 Induced Apoptosis of MM Cell Lines *in vitro* and *in vivo*

**Figure 2:**
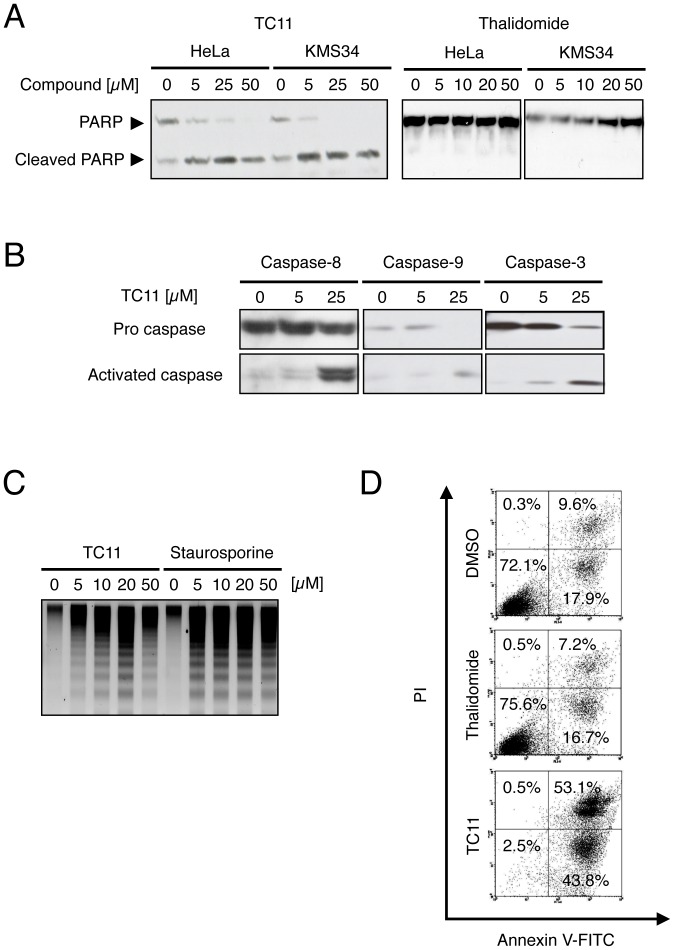
TC11 induces apoptosis of multiple myeloma cells in a caspase-dependent manner. (A) HeLa or KMS34 cells were treated with 0–50 µM TC11 or thalidomide for 24 h. The whole cell lysates were analyzed by Western blotting with anti-PARP antibody. (B) KMS34 cells were treated with 0, 5 or 25 µM TC11 for 6 h. The whole cell lysates were analyzed by Western blotting with anti-caspase-3, 8 or 9 antibody, respectively. (C) KMS34 cells were treated with 0–50 µM TC11 or staurosporin A for 6 h. After DNA extraction, 1% agarose gel electrophoresis was performed. (D) KMS34 cells were treated with 50 µM thalidomide or TC11. After 96 h, cells were stained with FITC-coupled annexin V and propidium iodide, and induction of apoptosis was evaluated by flow cytometry.

To examine whether TC11-induced apoptosis is dependent on the caspase pathway, we performed immunoblot analysis of lysates from TC11-treated KMS34 cells. After treatment with TC11 for 24 h, cleavage of PARP in both cell lines was detected, while treatment with thalidomide showed no effect (Figure 2A). Likewise, cleavage of procaspase-3, 8 and 9 (activated forms of caspase) was detected following treatment with TC11 (Figure 2B). DNA fragmentation was also observed in KMS34 cells treated with TC11 or staurosporine (Figure 2C). Besides these early-phase events (Fig. 2A–C), we also examined structural change of the cell membrane during apoptosis in the late phase (Fig. 2D). KMS34 cells were treated with 50 µM TC11 for 96 h. Annexin V-FITC and propidium iodide (PI) staining, followed by flow cytometric analyses, showed that TC11 treatment increased both Annexin V-positive/PI-negative and Annexin V-positive/PI-positive fractions (early and later apoptotic cells, respectively) of KMS34 cells, while thalidomide treatment did not. These results suggested that TC11 induced apoptosis depending on activation of caspase-3, 8 and 9.

**Figure 3:**
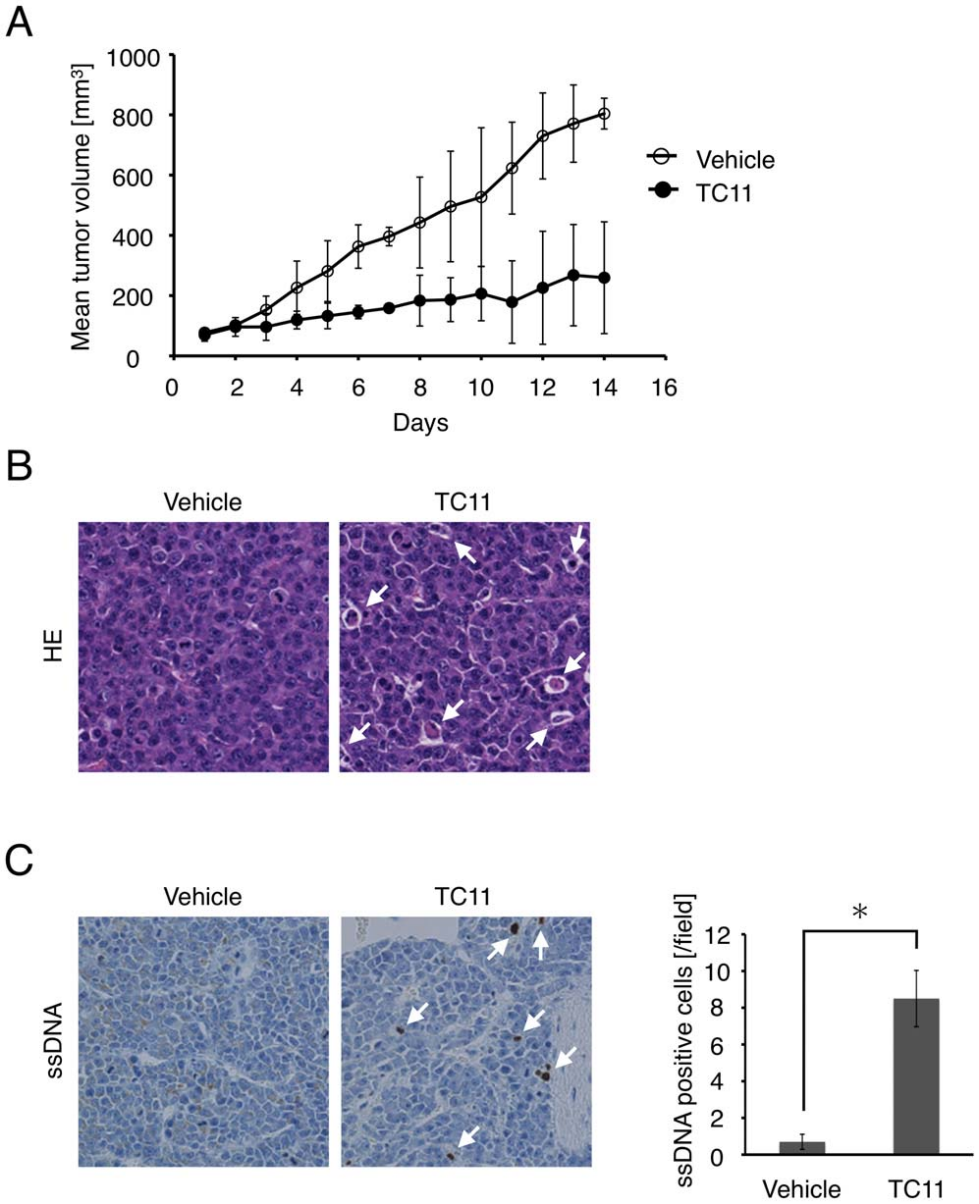
TC11 inhibits tumor cell growth and induces apoptosis *in vivo*. KMS34 (1.2×10^7^ cells) were injected intraperitoneally into lcr/scid mice and plasmacytoma was established. When the size of the tumor had reached 50 mm^3^ (day 1), 100 µL of TC11 (20 mg/kg) or vehicle alone (10% DMSO, 1% Tween 80-saline) was injected intraperitoneally into a mouse twice with a 3-day interval (i.e., injections were done on days 1, 2, 4, 5, 7, 8, 10, 11, 13 and 14). (A) The width and length of the plasmacytoma were measured and tumor volume was calculated (n = 7). (B) Sections were stained with hematoxylin and eosin (HE). Cells with aggregated chromatin are indicated by white arrows. (C) Apoptotic cell death was detected by immunohistochemical staining with anti-single-stranded DNA antibody. Apoptotic cells are indicated by white arrows. The plot on the right side shows that the difference in the number of ssDNA-positive cells between the vehicle and TC11 groups was statistically significant (Student’s *t*-test, P<0.002). For this plot, the number of ss DNA-positive cells was counted in two fields per tumor in five tumors (10 fields in all) in each group.

We next tested the anti-tumor activity of TC11 *in vivo*. KMS34 tumor xenografts (∼50 mm^3^) were treated with intraperitoneal injection of 20 mg/kg TC11 twice with a 3-day interval, followed by time-course analysis of tumor volume for 15 days (Figure 3A). After 7 and 14 days, TC11 showed significant tumor suppression (P<0.05). In the animal experiments, no mouse died and no macroscopic indications of TC11 toxicity were observed at autopsy. Hematoxylin-eosin staining of tumor tissue slices showed that cells with aggregated chromatin were increased in TC11-treated mice. Cytoplasm of these cells appeared round and little shrunken (Figure 3B). To examine whether these structural changes were caused by apoptosis, immunohistochemistry using anti-ssDNA antibody, which specifically detects fragmented single-strand DNA, was carried out. As shown in Figure 3C, DNA-fragmented cells detected by anti-ssDNA antibody were increased in the tumor tissue treated with TC11 (Figure 3C). These results suggested that TC11 likely induced apoptosis of KMS34 cell line tumor xenograft and exhibited anti-tumor activity *in vivo*.

### Identification of TC11-binding Proteins by mRNA Display

**Figure 4:**
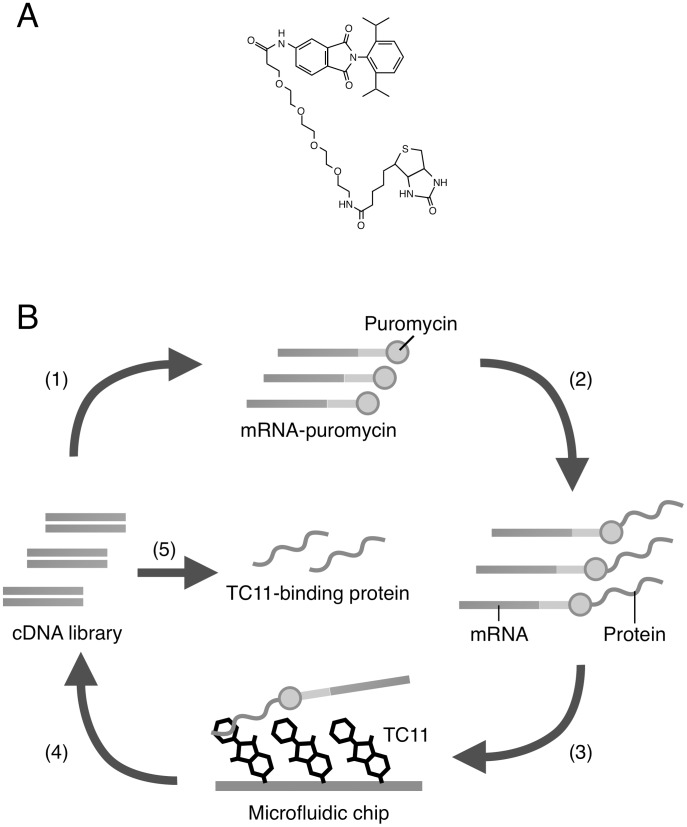
Schematic representation of *in vitro* selection of TC11-binding protein using mRNA display. (A) The chemical structure of biotinylated TC11. (B) A cDNA library derived from KMS34 cells was transcribed, ligated with PEG-Puro spacer (1) and *in vitro* translated (2) to form a protein-mRNA conjugates library. The library is injected into micro fluidic chip on which TC11 is immobilized (3) and unbound molecules were washed away. The bound molecules were eluted and their mRNA portion is amplified by RT-PCR (4). The resulted DNA can be used for the next of round and analyzed by cloning and sequencing. (See also Materials and Methods.).

To identify TC11-binding proteins, we used mRNA display [16], which is a powerful tool for *in vitro* selection of proteins that bind to various targets including small-molecular compounds. We first prepared a cDNA library derived from KMS34 cells, because our data suggested that KMS34 cells were the most sensitive to TC11. As a bait, biotinylated TC11 (Figure 4A) was immobilized on a microfluidic chip and selection of TC11-binding proteins were performed (Figure 4B). Although the 4-amino group of TC11, which was experimentally inferred to be critical for the activity, was biotinylated via a linker, the biotinylation hardly affect the antitumor activity (data not shown).

**Figure 5:**
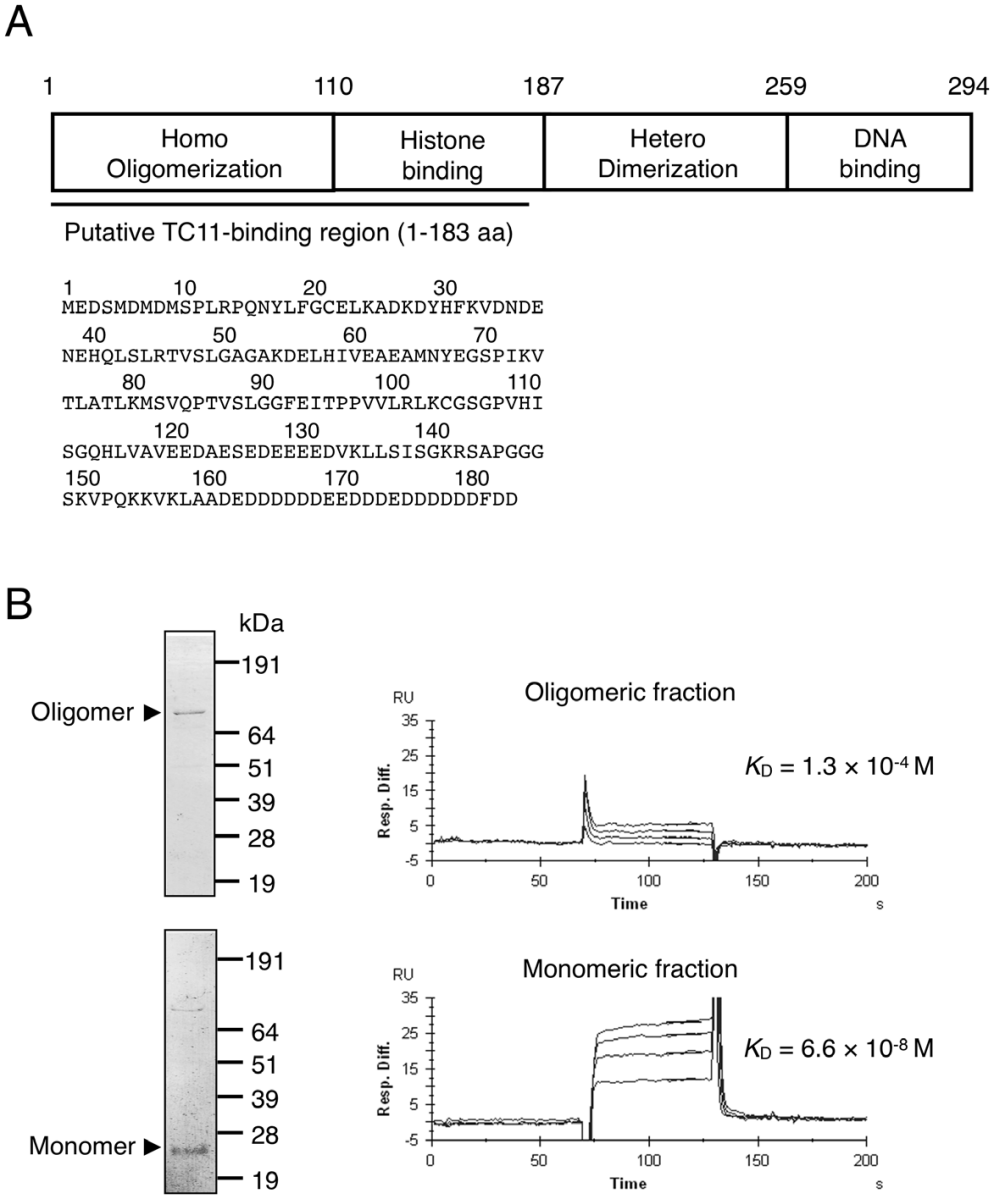
Monomeric NPM interacts with TC11. (A) The domain structure of NPM and the amino acid sequence predicted by NPM clone 1–183, which was identified as encoding a binding protein of TC11 by mRNA display using a cDNA library prepared from KMS34 cells. The underlined part indicates the region (1–183 a. a.) identified by mRNA display selection. The sequence indicated by the solid line corresponds to that encoded by the ORF of NPM cDNA. (B) Recombinant NPM_1–183_ was expressed in *E. coli* and fractionated into monomeric and oligomeric forms. Then, each fraction was subjected to 10% SDS-PAGE followed by CBB staining (left). A representative biosensorgram of NPM binding on SA sensor chips with immobilized biotinylated-TC11 is shown. The *K*_D_ values were determined (right).

Among 11 candidate TC11-binding proteins identified by mRNA display after 4 rounds of selection, we focused on nucleolar phosphoprotein nucleophosmin (NPM). Sequencing revealed that three selected NPM clones, designated 1–183 NPM, encoded the 183 NH2-terminal amino acids of NPM, which contains the oligomerization domain and a part of the histone binding domain (Figure 5A). The enrichment efficiency of the NPM clones was confirmed to be 10^4^-fold after 4 rounds of selection by RT-PCR. NPM is known to be a multifunctional protein involved in both tumorigenesis and tumor suppression [19], for example, it regulates cell proliferation and centrosome dupulication [20], [21] and stabilizes oncoprotein Myc [22] and tumor-suppressor protein p53 [23], [24]. Therefore, we hypothesized that NPM is involved in TC11-induced apoptosis of tumor cells.

To determine whether NPM interacts with TC11 directly, we examined the interaction between recombinant NPM and TC11. The initial *in vitro* binding assay between NPM and TC11 immobilized on beads revealed no interaction (data not shown). However, as NPM oligomerizes under native conditions [22] and its oligomerization domain may bind to TC11, we next performed gel filtration to separate oligomeric and monomeric forms of NPM, followed by surface plasmon resonance analysis of their affinity for TC11 (Figure 5B). These results indicate that the monomeric form of NPM binds to TC11 with a *K*_D_ value of 6.6×10^−8^ M, while the oligomer binds with a *K*_D_ value of 1.3×10^−4^ M, so that the monomeric form of NPM is the interactor with TC11.

### TC11 Inhibited Centrosomal Clustering and Thereby Induced Apoptosis

**Figure 6:**
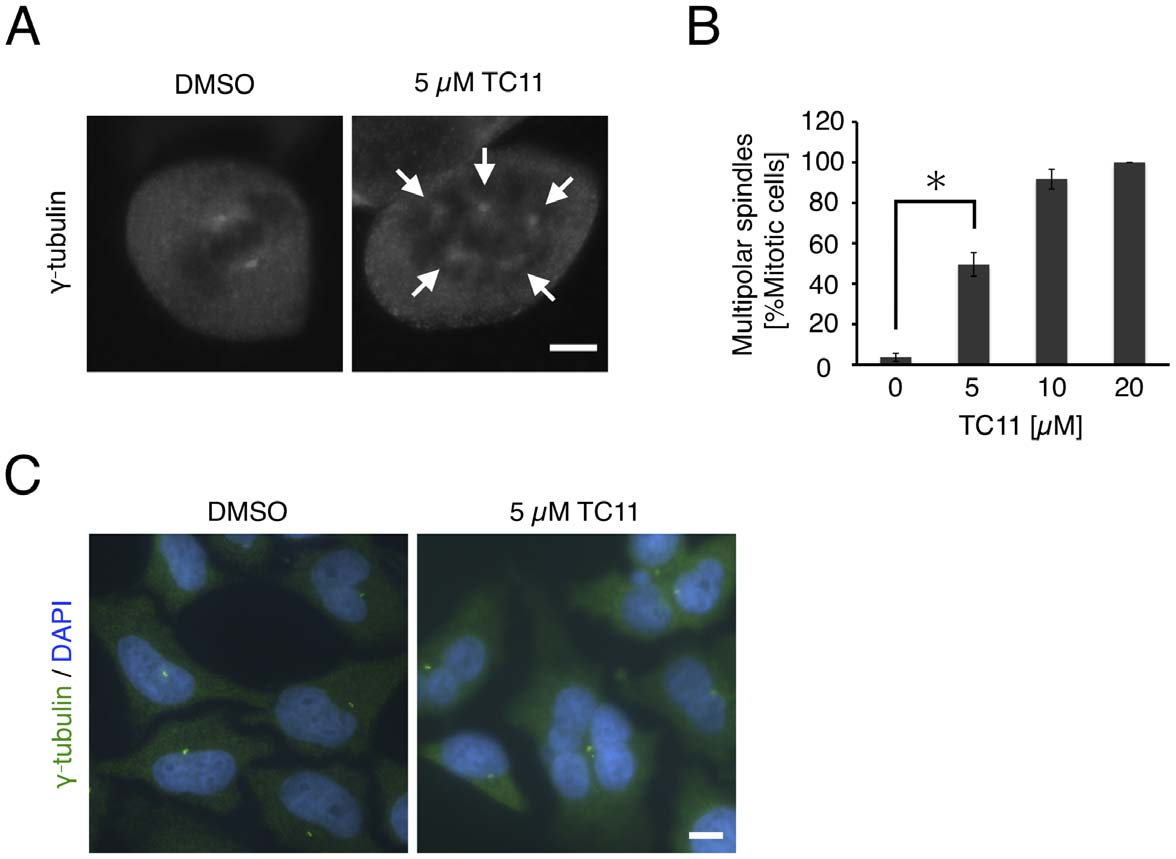
TC11 increases spindle multipolarity, resulting in multinucleation. HeLa cells were treated with 0–20 µM TC11 for 6 h. Then, immunofluorescence staining of γ-tubulin was performed. (A) Representative mitotic cell under the indicated conditions. (B) Mitotic cells with multipolar spindles were counted under the indicated conditions. At least 100 mitotic cells were counted per sample in three independent experiments. An asterisk denotes a statistically significant difference according to Student’s *t*-test (P<0.05). White arrows indicate centrosomes. (C) HeLa cells were treated with 5 µM TC11 for 24 h, followed by immunofluorescence staining with anti-γ-tubulin antibody (green). Nucleus was stained with DAPI (blue). Bar; 10 µm.

Although the oligomeric form of NPM regulates tumor-suppressor protein p53, and inhibition of NPM oligomerization results in activation of p53 leading to apoptosis of several tumor cell lines [23], [25], we used multiple myeloma cell line KMS34 in which p53 was inactivated in this study. Moreover, inhibition of NPM oligomerization and increase of p53 protein level were not observed in cell lines with active p53, such as HeLa cells (data not shown). Therefore, we could rule out the possibility that TC11-induced apoptosis of tumor cells is p53-pathway-dependent. It has been reported that NPM is localized on centrosomes during the mitotic phase of cells and regulates centrosomal duplication [17], [19]–[21]. Therefore, to examine the effect of TC11 on centrosomes, we performed immunofluorescence staining and fluorescence microscopic observation. In order to carefully observe intra-nuclear structures of TC11-treated cells, and due to difficulty of gene transfer to multiple myeloma cell lines, we chose HeLa cells. HeLa cells were incubated with 0–20 µM TC11 for 6 h, followed by staining with anti-γ-tubulin antibody. Many mitotic-phase cells with multipolar spindles (>2) were observed in the case of treatment with TC11, while many mitotic cells with bipolar spindles were observed in the case of treatment with DMSO (Figure 6A). Quantification of multipolarity indicated that induction of multipolar mitoses was TC11 concentration-dependent (Figure 6B). Several studies have shown that multipolarity occurs due to inhibition of centrosomal clustering [26], [27]. Thus, TC11 may inhibit centrosomal clustering and induce multipolar spindles in mitotic cells.

Additionally, we found that mitosis of cells with multipolar spindles resulted in multinucleated cells. After 24 h treatment of HeLa cells with TC11, most interphase cells had multiple nuclei, while control cells had a single nucleus (Figure 6C). The former cells may occur as a result of division of cells with multipolar spindles. It was recently reported that cells with multiple nuclei have poor viability and undergo apoptosis [28]. Therefore, we considered that TC11-induced apoptosis would occur through this mechanism.

### NPM Knockdown Induced Multipolar Spindles and Apoptosis

**Figure 7:**
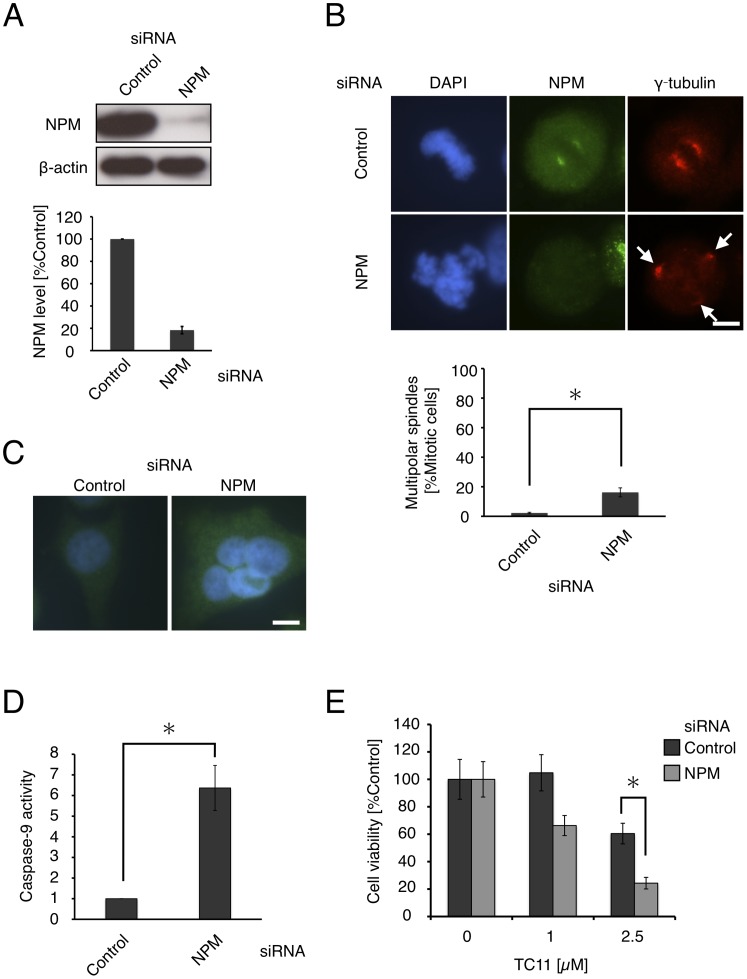
NPM knockdown induces multipolar spindles. HeLa cells were transfected with siRNA for luciferase (control) or NPM. (A) After 48 h, the whole cell lysates were analyzed by western blotting with antibody against NPM (left) and the band intensities were quantified (right). (B) Immunofluorescence with anti-NPM (green) and anti-γ-tubulin (red) antibody was performed (left). White arrows indicate centrosomes. At least 50 mitotic cells were counted in three independent experiments. The ratio of cells with multipolar spindles under the indicated conditions was quantified (below). (C) Representative multinucleated cell under the indicated conditions. (D) After 72 h, caspase-9 activity was determined using luminescence-based assay. (E) At 48 h after siRNA transfection, the cells were treated with 0–2.5 µM TC11 and then incubated for a further 72 h. Cell viability was determined by means of WST-1 assay in three independent experiments. An asterisk denotes a statistically significant difference according to Student’s *t*-test (P<0.05). Bar; 10 µm.

To investigate whether NPM is related to the multipolarity of mitotic cells, we performed a NPM knockdown experiment. HeLa cells were transfected with siRNA for NPM and after 48 h, NPM protein levels were confirmed to be repressed to <20% (Figure 7A). After siRNA transfection, we performed immunofluorescence staining with anti-γ-tubulin antibody. We found that mitotic cells with multipolar spindles amounted to 16% of NPM-depleted cells, but only 2% of control cells (Figure 7B). Additionally, multinucleated cells were observed only among cells transfected with siRNA for NPM (Figure 7C). To examine whether knockdown of NPM results in apoptosis, we also performed caspase-9 activity assay. Caspase-9 activity was 6-fold higher in the case of NPM-depleted cells than control cells (Figure 7D). We finally examined whether the inhibitory activity of TC11 on cell proliferation involves NPM. HeLa cells were treated with TC11 after knockdown of NPM and the cell viability was determined by means of WST-1 assay (Figure 7E). We found that TC11 showed more potent cytotoxicity against NPM-depleted HeLa cells than against control cells, indicating that down-regulation of NPM increases the sensitivity of HeLa cells to TC11. These results suggested that inhibition of NPM function results in multipolarity of mitotic cells, leading to apoptosis.

## Discussion

Although the prognosis of multiple myeloma has been improved by the introduction of drugs such as thalidomide, lenalidomide and bortezomib, high-risk patients tend to show a poor response. We considered that KMS34 cell line would be a good model for high-risk cases with t(4;14) or del 17. Therefore, we used KMS34 cells to screen for compounds able to induce apoptosis of high-risk myeloma cells. Among the 29 phthalimide derivatives examined, TC11 showed significant anti-tumor activity against multiple myeloma KMS34 cells both *in vitro* and *in vivo*, while existing drugs such as thalidomide and lenalidomide had little or no effect *in vitro* (Fig. S2 and unpublished data). These results suggest that TC11 would be effective against multiple myeloma with high-risk chromosomal changes, t(4;14) and del17 (where the p53 tumor suppressor gene is located).

We found that TC11 induced apoptosis of tumor cells through inhibition of centrosomal clustering. Rebacz *et al.* recently reported that griseofulvin also inhibits centrosomal clustering [27]. Both TC11 and griseofulvin induce caspase-3, 8 and 9 activation, followed by cleavage of PARP. Although their action mechanisms for inducing apoptosis seem very similar, TC11 is a more potent inducer of multipolarity and apoptosis. Furthermore, multinucleated cells were observed following treatment with TC11 for 24 h, probably due to aberrant cell division, because it was previously reported that multinucleated cells resulted from aberrant cell division of cells with multipolar spindles [29]. Although the molecular mechanism that triggers apoptosis after aberrant cell division is unknown, another study also found that multinucleated cells show poor survival capability [28]. Thus, inhibition of centrosomal clustering may play a major role in TC11-induced apoptosis.

We identified NPM as a candidate target protein of TC11 for inducing apoptosis and found that the monomeric form of NPM, but not oligomeric NPM, bound tightly to TC11. Since the putative TC11-binding region of NPM includes its oligomerization domain, and it has been reported that inhibiting oligomerization of NPM leads to apoptosis [30], [31], we hypothesized that TC11 inhibits oligomerization of NPM. However, inhibition of the oligomerization could not be confirmed with native-PAGE (data not shown).

Our data suggested that NPM may be involved in the appearance of multipolar spindles in mitotic cells. NPM is known to regulate centrosomal duplication during the mitotic phase of cell division. Although a recent study did not identify NPM as a protein that was required for centrosomal clustering [32], siRNA-induced knockdown of the NPM gene has been reported to induce multipolar spindles in HeLa cells [33], and we also confirmed this result in the present work. Wang *et al.* previously reported that preventing export of NPM to cytoplasm from nucleus through disruption of the NPM-CRM1 complex with the CRM1 inhibitor leptomycin B (LMB) resulted in dissociation of NPM from centrosomes, and thereby led to multipolar spindle formation [34]. In conflict with that report, our immunofluorescence experiments indicated that NPM in both TC11 and LMB-treated cells is localized on centrosomes (Figure S3). In support of our finding, a recent study by Rousselet indicated that LMB does not disturb localization of NPM to centrosomes or affect centrosomes numbers [35]. Therefore, it appears that interaction of TC11 with NPM may inhibit its centrosomal-regulatory function without affecting its localization on centrosomes. Little is known about the region of NPM that is required to regulate centrosomal duplication or whether NPM exists as oligomer when it localizes to centrosomes. Moreover, NPM interacts with many kinds of proteins such as Myc, p53, MDM2 and so on [22]–[24], [36]. It is possible that other NPM-interacting protein(s) are involved in centrosomal clustering. Further study is needed to establish the significance of TC11-NPM interaction for the anticancer effect of TC11.

We also found that thalidomide did not inhibit centrosomal clustering (Figure S4). It was previously reported that thalidomide interferes with tumor angiogenesis. The difference in action mechanism between thalidomide and TC11 is presumably related to the difference in their chemical structure. It is noteworthy that TC11 showed anti-tumor activity against multiple myeloma KMS34 cells that were resistant to thalidomide (Figure S2) and lenalidomide (unpublished data).

Inatsuki *et al.* previously reported that a compound with the same structure as TC11 inhibited tubulin polymerization [37]. In our mRNA display experiments, α-tubulin was identified as TC11-binding protein, as well as NPM (data not shown). Thus, there may be a possibility that TC11-induced apoptosis is triggered by inhibition of tubulin polymerization. However, colchicine, which inhibits tubulin polymerization and induces apoptosis, did not induce multipolarity of mitotic cells (data not shown) [38]. Moreover, our data showed that repression of NPM leads to apoptosis and increased sensitivity to TC11. Further experiments, for example with NPM mutants that do not bind to TC11, might be helpful to establish the relationship between NPM and TC11-induced apoptosis. Therefore, if binding of TC11 to α-tubulin does induce apoptosis by inhibition of tubulin polymerization, we consider that this occurs independently of the pathway that relates to inhibition of centrosomal clustering.

In conclusion, we have identified TC11 as a potent suppressor of proliferation of multiple myeloma cells with high-risk chromosomal or genetic changes, both *in vitro* and *in vivo*. Our results suggest that TC11 inhibits the centrosomal-regulatory function of NPM, thereby inducing multipolar mitotic cells, which undergo apoptosis. Although further work is required to fully establish the role of NPM in apoptosis, NPM may become a novel target for development of antitumor drugs active against myeloma cells.

## Materials and Methods

### Cell Lines

Multiple myeloma (MM) cell lines (KMM1, KMS11, KMS26, KMS27, KMS34 and RPMI8226) were established by T Otsuki (Kawasaki Medical College, Kurashiki, Japan) from Japanese patients [39] and were maintained in RPMI1640 medium with 10% fetal bovine serum and 1% penicillin/streptomycin. HeLa cells (RIKEN Cell Bank, 2002) were maintained in DMEM with 10% fetal bovine serum and 1% penicillin/streptomycin. The identification of cell lines was performed based on an STR Multiplex method that uses 9 different loci: D5S818, D13S317, D7S820, D16S539, vWA, TH01, Amelogenin, TPOX and CSF1PO (Powerplex 1.2 system, Promega Corporation) in 2011.

### Compounds

The compounds listed in Figs. S1 and S2 were prepared in 50–95% yields by refluxing a mixture of phthalic acid anhydride derivatives and appropriate amines in acetic acid for several hours. Phthalimide derivatives with an amino group, such as TC1-16 and TC11-42, were synthesized from phthalic acid anhydride with a nitro group and appropriate amines, followed by catalytic hydrogenation under a hydrogen atmosphere. The chemical structures of synthetic compounds were confirmed by ^1^H-NMR spectroscopy and mass spectrometry.

### Compound Screening

Synthetic compounds were dissolved in DMSO to make 20 mM stock solutions. The stock solutions were diluted to 0.5–50 µM in medium and distributed into 96-well plates. Then, MM cells (1×10^4^ cells/well) were seeded in each well and incubated for 72 h. The number of viable cells was determined with the reagent WST-1 (Roche, Basel, Switzerland) according to the manufacturer’s instructions.

### Immunoblot Analysis

Cells were treated with various concentrations of TC11, TC13 or thalidomide for 6–48 h, followed by lysis in RIPA buffer (50 mM Tris-HCl pH 7.6, 150 mM NaCl, 1 mM EDTA, 0.5% sodium deoxycholate, 0.05% SDS, 1% NP-40) containing protease inhibitor cocktail (Nacalai Tesque, Kyoto, Japan). Protein concentrations were determined using BCA protein assay kit (Thermo Scientific, Waltham, MA) and 20 µg of protein was loaded on a 10% or 15% SDS-PAGE gel and analyzed with antibodies against caspase-3, caspase-8, caspase-9, PARP (Cell Signaling Technology, Beverly, MA), or NPM (Sigma, St. Louis, MO, USA). The blots were developed using ECL chemi-luminescence reagents (GE Healthcare, Waukesha, WI).

### DNA Fragmentation Assay

KMS34 cells were treated with various concentrations of TC11 for 6 h. The cells were lysed with lysis buffer (10 mM Tris-HCl pH 7.4, 10 mM EDTA, 0.2% Triton X-100), followed by incubation on ice for 15 min. The solution was centrifuged at 10,000*g* for 20 min and the supernatant was treated with 100 µg/ml RNase A. The resulting solution was purified with MaXtract (Qiagen, Valencia, CA) according to the manufacturer’s instructions, followed by isopropanol precipitation. DNA laddering was detected by EtBr staining after agarose gel electrophoresis.

### Flow Cytometric Analysis

KMS26, KMS27 and KMS34 cells (2×10^5^ cells, respectively) were incubated with 50 µM thalidomide or TC11 for 96 h. The cells were stained with an Annexin V-FITC kit (Bender Medsystems, Vienna, Austria) and Annexin V-positive cells were quantified with a Becton Dickinson FACSCalibur system (Becton Dickinson, San Jose, CA).

### *In vivo* Tumor Growth Assay

All of the animal experiments were approved by the Ethics Committee for Animal Experiments at Keio University School of Medicine (Approval no. 09118-0). *In vivo* tumor-inhibitory activity assay was performed as previously described [40] with several modifications. Briefly, 3×10^7^ KMS34 cells were subcutaneously inoculated into 5-week-old male lcr/scid mice (CLEA, Tokyo, Japan) and plasmacytoma developed in 4 to 6 weeks. TC11 was dissolved in DMSO (Sigma) at concentration of 20 mg/mL and then diluted with 5% carboxymethyl cellulose-saline solution to 2.5 mg/mL. Tumor volume was calculated according to the following formula: width×length^2^×0.52 [40]. Differences in the size of tumors on days 7, 10 and 14 were compared and evaluated by means of Student’s *t* test. P<0.05 was considered to indicate statistical significance.

### Histopathologic Examination

Histopathologic analysis was performed as previously described [40] with several modifications. When the subcutaneous tumors reached 50 mm^3^, 5% carboxymethylcellulose or 20 mg/kg TC11 was injected intraperitoneally twice with an interval of 3 days. After 14 days, the mice were killed and the tumors were isolated. Tumor samples were fixed with 10% formalin and embedded in paraffin. Sections were stained with hematoxylin and eosin. Apoptotic cell death was determined with anti-single-stranded DNA antibody (DakoCytomation, Carpinteria, CA).

### mRNA Display Selection on a Microfluidic Chip

The affinity selection of target proteins of TC11 was performed by combined mRNA display with a microfluidic system [41]. Total RNA from KMS34 cells was extracted with an RNeasy mini kit (Qiagen), followed by purification with a mTRAP mRNA isolation kit (Active Motif, Carlsbad, CA). Thereafter, preparation of a cDNA library was performed as previously described [42]. The resulting cDNA library derived from KMS34 cells was transcribed using a RiboMAX large-scale RNA production system-SP6 (Promega, Madison, WI). The resulting RNA was purified with the RNeasy mini kit and ligated with a PEG-puromycin spacer [p(dCp)_2_-T(fluorescein)p-PEGp-(dCp)_2_-puromycin] [16] using T4 RNA ligase (Takara, Otsu, Japan) at 15°C for 15 h. The resulting RNA-PEG-puromycin library was purified with the RNeasy mini kit. *In vitro* translation was performed using wheat germ cell-free extract (Zoegene). The reaction mixture was subjected to gel filtration on Sephadex G200 (GE Healthcare) using a 0.8×4 cm column (Bio-Rad, Richmond, CA) with TBS containing 10 mM EDTA, and 2-drop fractions were collected. The fluorescence of mRNA-displayed proteins in eluate fractions was monitored with a Multi-detection microplate reader Powerscan HT (Dainippon Pharmaceutical). The biotinylated TC11 was immobilized on SA sensor chips (GE Healthcare) by passing HBS-EP buffer at a flow rate of 10 µL/min on a Biacore 3000 instrument (GE Healthcare). Amounts of biotinylated TC11 immobilized on the chips in flow cells Fc-1, Fc-2, Fc-3 and Fc-4 were 203, 205, 206 and 207 RU, respectively. The 4th to 7th fractions containing mRNA-displayed proteins were diluted with HBS-EP buffer to 300 µL and injected onto the antigen-immobilized sensor chip. The selection experiments were performed at 25°C with the Biacore 3000 using HBS-EP buffer at a flow rate of 20 µL/min. After association for 250 s and dissociation for 1000 s, the sensor surfaces were washed once with HBS-EP buffer for 600 s. The bound molecules were eluted competitively from the sensor surface with 7 µL of 200 µM free TC11 for 600 s. Then, recovered solution was subjected to RT-PCR in a total volume of 100 µL containing 1 mM dNTPs, 50 mM Tris-HCl, pH 8.0, 75 mM KCl, 3 mM MgCl_2_, 10 mM dithiothreitol, 200 U of RNase inhibitor, 500 U of ReverTraAce (Toyobo), and 50 pmol 3RV30 primer (5′-TTTTTTTTCTTGTCGTCATCGTCCTTGTAG-3′), at 50°C for 30 min and heated at 99°C for 5 min. The RT product was amplified by PCR with KOD-plus DNA polymerase using primers Gsp6Omega F (5′-GGAAGATCTATTTAGGTGACACTATAGAACAACAACAACAACAAACAACAACAAAATG-3′) and 3RV30 [24–36 cycles of 30 s at 94°C, 30 s at 58°C, and 2 min at 68°C]. The resulting DNA was purified using a Wizard PCR preps DNA purification kit (Promega). Finally, selected DNAs were cloned using a TOPO TA cloning kit (Invitrogen) and sequenced with a CEQ 2000 DNA analysis system (Beckman Coulter, Brea, CA). Genetyx-mac 13.0.10 sequence analysis software and ClustalX 1.83 were used for alignment and sequence manipulations.

### Surface Plasmon Resonance Analysis

Binding kinetics was determined by surface plasmon resonance (SPR) analysis with a Biacore 3000 (GE Healthcare). All experiments were performed at 25°C using HBS-EP buffer. Biotinylated TC11 was immobilized onto the SA sensor chip (GE Healthcare). The measurements were performed under conditions of 276 resonance units of the ligand and at a flow rate of 30 µL/min. To determine dissociation constants, four different concentrations of partially purified monomeric NPM and oligomeric NPM were injected. The injection period for association was 60 s. After each measurement, the chip surface was regenerated with 10 µL of Glycine 2.0 (GE Healthcare). The binding data were analyzed with the steady-state affinity model in the BIAevaluation software ver. 4.1 (GE Healthcare).

### Immunofluorescence Assay and TUNEL Staining

HeLa cells on coverslips were treated with 5–20 µM TC11 for 6 h and then fixed with cold methanol and blocked with 1% BSA in PBS for 30 min. The sample was stained with antibody against NPM (Invitrogen) or γ-tubulin followed by Alexa488-conjugated anti-mouse IgG (Invitrogen) or CF568-conjugated anti-rabbit IgG (Biotium, Hayward, CA), respectively.

### siRNA Transfection

HeLa cells (5×10^4^ cells) were transfected with NPM siRNA oligonucleotides (5′-ATGGAATGTTATGATAGGACA-3′) or luciferase siRNA oligonucleotides (5′-CGTACGCGGAATACTTCGA-3′) (Invitrogen) using a Neon transfection system (Invitrogen) according to the manufacturer’s instructions.

### Measurement of Caspase-9 Activity

Caspase-9 activity was measured with a luminescence-based assay kit, Caspase-Glo 9 Assay (Promega). After having been transfected with siRNA, 1×10^4^ HeLa cells were cultured in 96-well plates for 72 h. At the end of incubation, 100 µl of assay reagent was added and incubation was continued for 1 h at room temperature. Luminescence was measured using a Microplate luminometer (Promega).

## Supporting Information

Figure S1 **Chemical structures of phthalimide derivatives.**
(TIF)

Figure S2 **Screening of compound that inhibit multiple myeloma cell lines.** (A) KMS34 cells (1×10^4^ cells/well) in 96-well plate were incubated with 50 µM of each compound from phthalimide derivatives library for 0, 24, 48 or 72 h. Then cell viability was determined with WST-1 assay according to instructions provided by the manufacturer. (B) KMS34 or RPMI8226 cells were treated with 0, 5 or 50 µM TC11 or TC15 for 6 h, respectively. The whole cell lysates were analyzed by Western blot with anti-PARP antibody.
(TIF)

Figure S3 **TC11 or LMB does not affect localization of NPM on centrosome.** (A) HeLa cells were treated with 5 µM TC11 or 100 nM LMB for 6 h. Then, immunofluorescence staining of NPM (green) or γ-tubulin (red) was performed. Representative mitotic cells under the indicated conditions are shown. White arrowheads and arrows indicate NPM and centrosomes, respectively. White arrow indicates centrosome. Bar; 10 µm.
(TIF)

Figure S4 **Thalidomide does not induce multipolarity of mitotic cells.** HeLa cells were treated with 10 or 20 µM thalidomide for 6 h. Then, immunofluorescence staining of γ-tubulin was performed. At least 50 mitotic cells were counted in three independent experiments. The ratio of cells with multipolar spindles under the indicated conditions was quantified.
(TIF)
