# Supplementary figures and images for: A Phthalimide Derivative That Inhibits Centrosomal Clustering Is Effective on Multiple Myeloma
Source: PLoS One. 2012 Jun 25;7(6):e38878. doi: 10.1371/journal.pone.0038878 (PMC3382596; doi:10.1371/journal.pone.0038878)

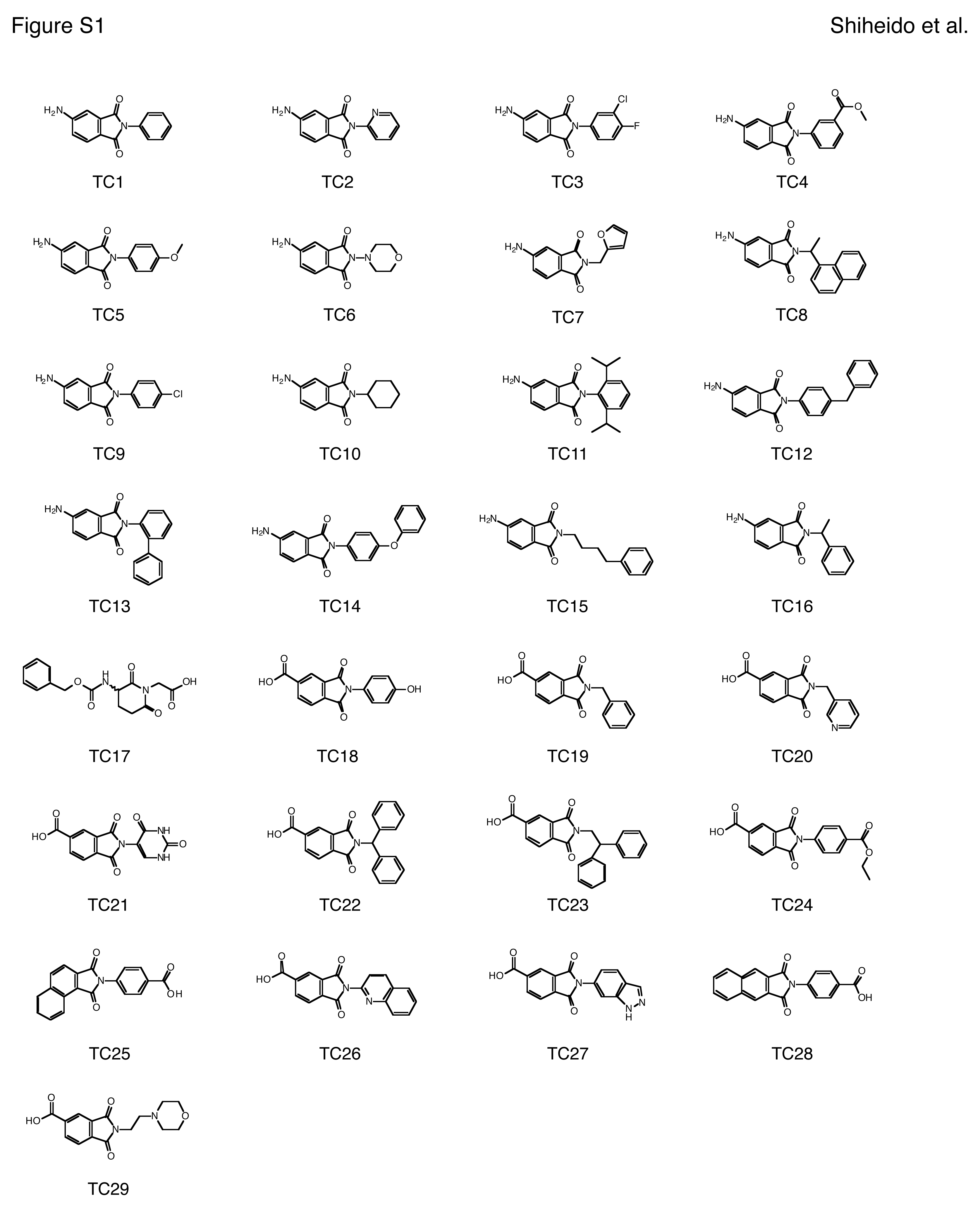

Supplement: Figure S1 — Chemical structures of phthalimide derivatives. (TIF) [file pone.0038878.s001.tif]

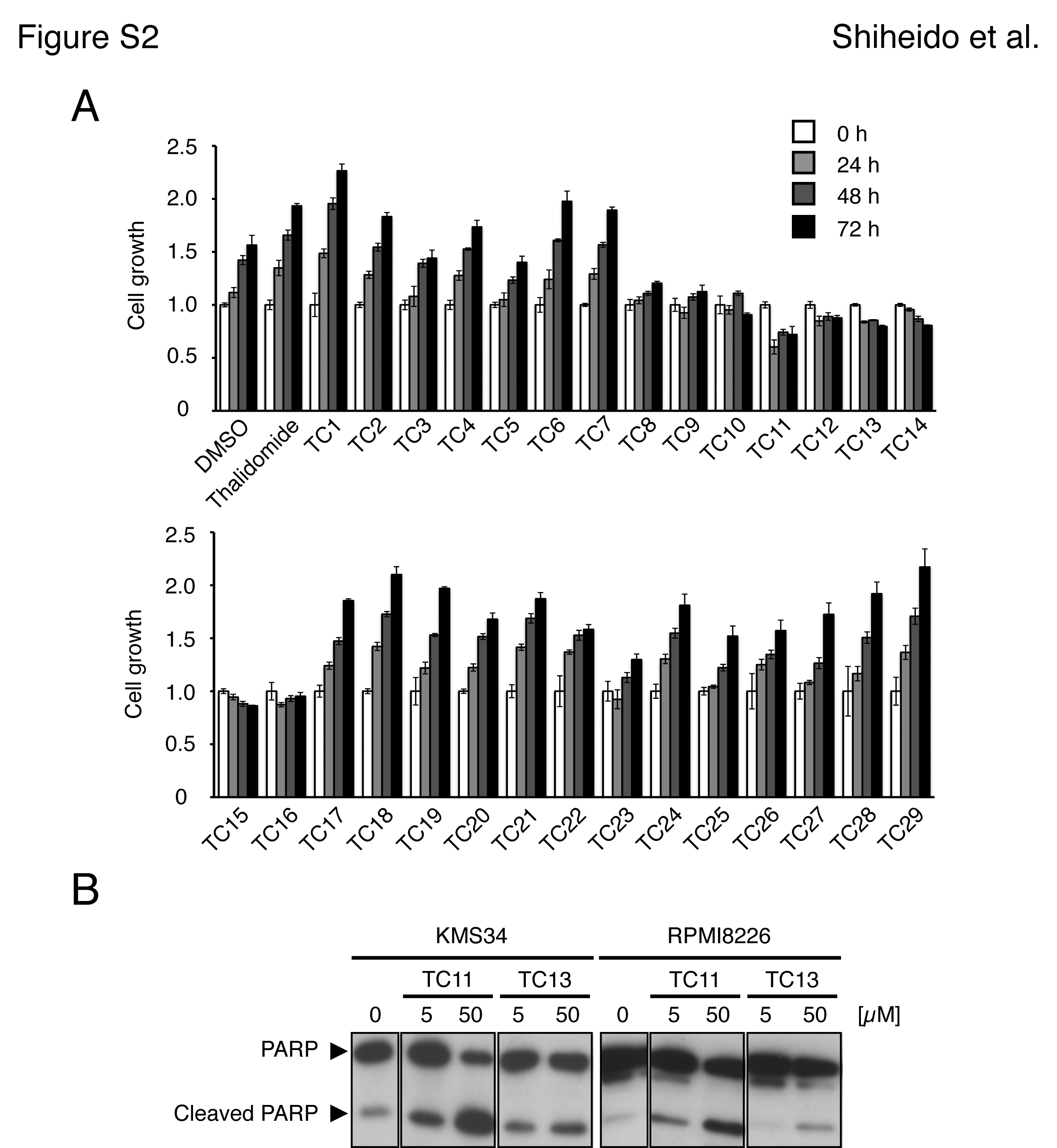

Supplement: Figure S2 — Screening of compound that inhibit multiple myeloma cell lines. (A) KMS34 cells (1×10^4^ cells/well) in 96-well plate were incubated with 50 µM of each compound from phthalimide derivatives library for 0, 24, 48 or 72 h. Then cell viability was determined with WST-1 assay according to instructions provided by the manufacturer. (B) KMS34 or RPMI8226 cells were treated with 0, 5 or 50 µM TC11 or TC15 for 6 h, respectively. The whole cell lysates were analyzed by Western blot with anti-PARP antibody. (TIF) [file pone.0038878.s002.tif]

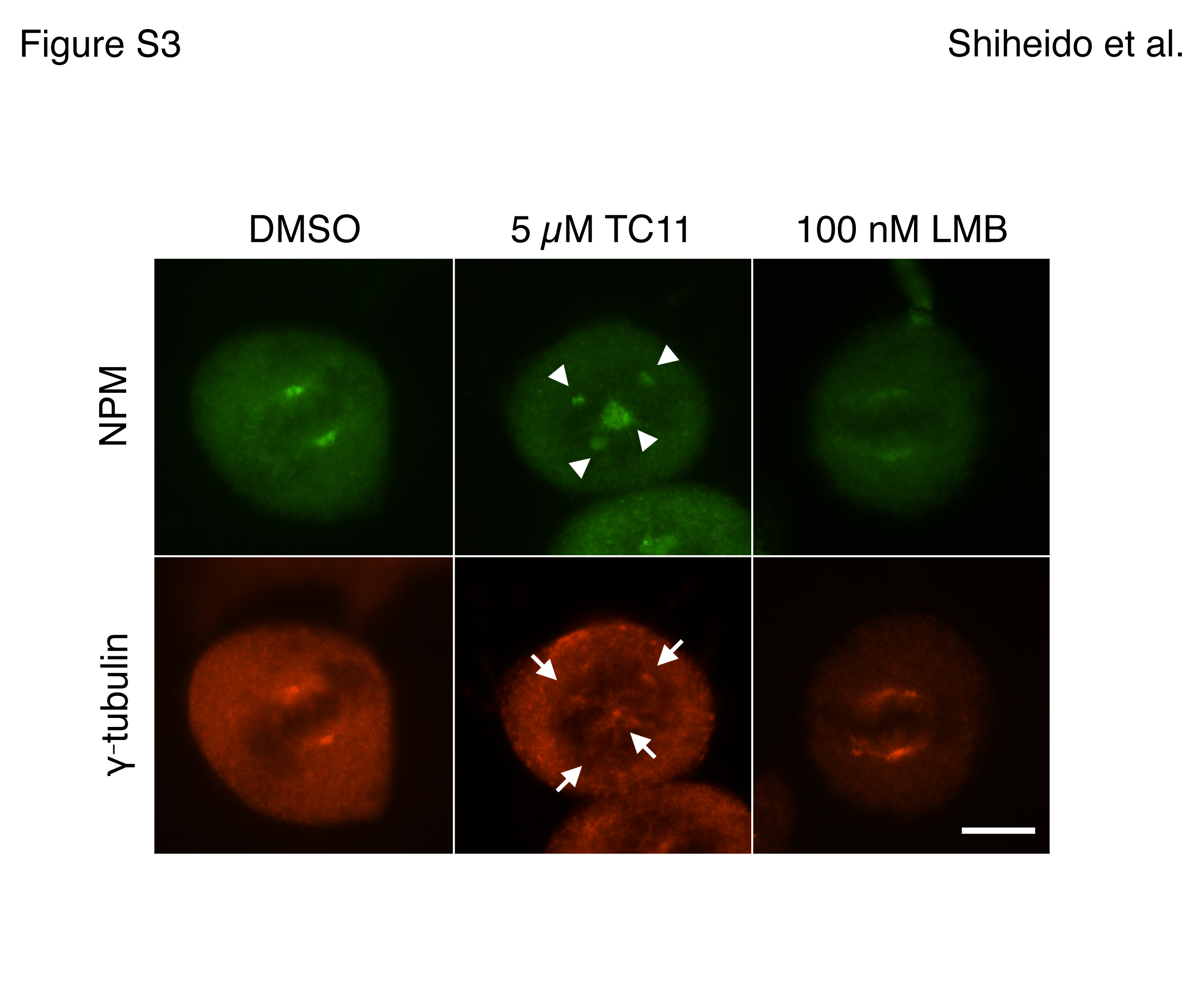

Supplement: Figure S3 — TC11 or LMB does not affect localization of NPM on centrosome. (A) HeLa cells were treated with 5 µM TC11 or 100 nM LMB for 6 h. Then, immunofluorescence staining of NPM (green) or γ-tubulin (red) was performed. Representative mitotic cells under the indicated conditions are shown. White arrowheads and arrows indicate NPM and centrosomes, respectively. White arrow indicates centrosome. Bar; 10 µm. (TIF) [file pone.0038878.s003.tif]

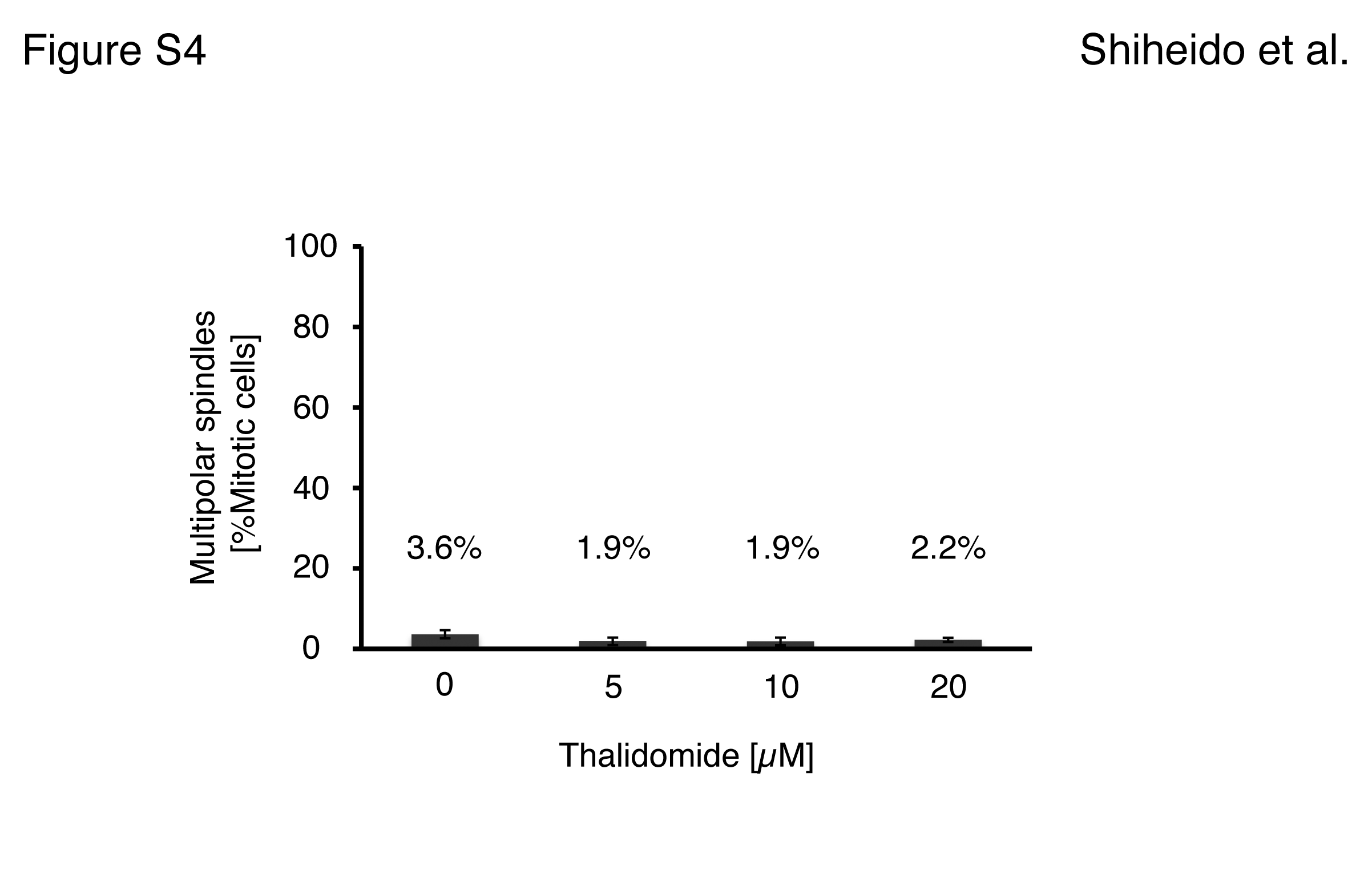

Supplement: Figure S4 — Thalidomide does not induce multipolarity of mitotic cells. HeLa cells were treated with 10 or 20 µM thalidomide for 6 h. Then, immunofluorescence staining of γ-tubulin was performed. At least 50 mitotic cells were counted in three independent experiments. The ratio of cells with multipolar spindles under the indicated conditions was quantified. (TIF) [file pone.0038878.s004.tif]
